# LEDGF/p75 Overexpression Attenuates Oxidative Stress-Induced Necrosis and Upregulates the Oxidoreductase ERP57/PDIA3/GRP58 in Prostate Cancer

**DOI:** 10.1371/journal.pone.0146549

**Published:** 2016-01-15

**Authors:** Anamika Basu, Christina K. Cajigas-Du Ross, Leslimar Rios-Colon, Melanie Mediavilla-Varela, Tracy R. Daniels-Wells, Lai Sum Leoh, Heather Rojas, Hiya Banerjee, Shannalee R. Martinez, Stephanny Acevedo-Martinez, Carlos A. Casiano

**Affiliations:** 1 Center for Health Disparities and Molecular Medicine, Department of Basic Sciences, Loma Linda University School of Medicine, Loma Linda, California 92350, United States of America; 2 Department of Pathology and Human Anatomy, Loma Linda University School of Medicine, Loma Linda, California 92350, United States of America; 3 Novartis Pharmaceutical Oncology, East Hanover, New Jersey 08807, United States of America; 4 Department of Medicine, Loma Linda University School of Medicine, Loma Linda, California 92350, United States of America; Hormel Institute, University of Minnesota, UNITED STATES

## Abstract

Prostate cancer (PCa) mortality is driven by highly aggressive tumors characterized by metastasis and resistance to therapy, and this aggressiveness is mediated by numerous factors, including activation of stress survival pathways in the pro-inflammatory tumor microenvironment. LEDGF/p75, also known as the DFS70 autoantigen, is a stress transcription co-activator implicated in cancer, HIV-AIDS, and autoimmunity. This protein is targeted by autoantibodies in certain subsets of patients with PCa and inflammatory conditions, as well as in some apparently healthy individuals. LEDGF/p75 is overexpressed in PCa and other cancers, and promotes resistance to chemotherapy-induced cell death via the transactivation of survival proteins. We report in this study that overexpression of LEDGF/p75 in PCa cells attenuates oxidative stress-induced necrosis but not staurosporine-induced apoptosis. This finding was consistent with the observation that while LEDGF/p75 was robustly cleaved in apoptotic cells into a p65 fragment that lacks stress survival activity, it remained relatively intact in necrotic cells. Overexpression of LEDGF/p75 in PCa cells led to the upregulation of transcript and protein levels of the thiol-oxidoreductase ERp57 (also known as GRP58 and PDIA3), whereas its depletion led to ERp57 transcript downregulation. Chromatin immunoprecipitation and transcription reporter assays showed LEDGF/p75 binding to and transactivating the ERp57 promoter, respectively. Immunohistochemical analysis revealed significantly elevated co-expression of these two proteins in clinical prostate tumor tissues. Our results suggest that LEDGF/p75 is not an inhibitor of apoptosis but rather an antagonist of oxidative stress-induced necrosis, and that its overexpression in PCa leads to ERp57 upregulation. These findings are of significance in clarifying the role of the LEDGF/p75 stress survival pathway in PCa.

## Introduction

Prostate cancer (PCa) is the second leading cause of cancer deaths among men in the United States, affecting disproportionately African American men compared to other racial/ethnic groups [1]. PCa initiation and progression has been linked to chronic inflammation and increased oxidative damage in this gland [2,3]. As a mechanism of survival in this stressful environment, PCa cells activate stress survival pathways that promote tumor aggressive properties, including resistance to cell death and chemotherapy [4–6]. Lens epithelium-derived growth factor of 75 kD (LEDGF/p75) is an emerging oncoprotein that promotes mammalian cell survival in the presence of environmental stressors that increase cellular oxidative damage [7–14]. Also known as transcription co-activator p75, PC4 and SFRS1 interacting protein (PSIP1), and dense fine speckled autoantigen of 70 kD (DFS70), this multifunctional protein has gained relevance in the study of cancer, HIV-AIDS, autoimmunity, and eye disease (reviewed in refs. [9,10]). As the key cellular co-factor for HIV integration into host chromatin, LEDGF/p75 has attracted considerable attention during the past decade, and vigorous efforts are currently under way to target this protein for the treatment of HIV-AIDS [15].

The emerging role of LEDGF/p75 as a stress oncoprotein has been uncovered by several studies from our group and others documenting its overexpression in diverse human cancer types, and its ability to induce features associated with tumor aggressiveness in cancer cells [10–14,16–19]. In addition, LEDGF/p75 is aberrantly expressed in human leukemias, and interacts with the Menin-MLL (mixed leukemia lineage) transcription complex to activate the expression of cancer-associated genes and leukemogenesis [20,21]. The potential of LEDGF/p75 as a promising target for cancer treatment has been highlighted by studies showing that its inhibition or downregulation attenuates the aggressive properties of cancer cells [14,17,19,21,22].

Our group and others demonstrated previously that LEDGF/p75 is the target of an autoantibody response in a subset of PCa patients, as well as in apparently healthy individuals and patients with diverse chronic inflammatory conditions ([23], also reviewed in refs. [9,10]). We also reported that LEDGF/p75 is overexpressed in prostate tumors and that this overexpression promotes PCa cell resistance to caspase-independent lysosomal cell death induced by the taxane drug docetaxel (DTX), the gold standard for PCa chemotherapy [11,13,23]. Interestingly, LEDGF/p75 upregulation occurs naturally during the selection of DTX-resistant PCa cells [24]. In concordance with these observations, several studies showed that LEDGF/p75 overexpression in cancer cells promotes resistance to drugs that induce oxidative DNA damage and lysosomal cell death [12–14,18,25], leading one group to refer to this protein as a “guardian of lysosomal stability in human cancer” [14]. The stress protective functions of LEDGF/p75 appear to be mediated by its ability to participate in DNA repair and transcriptionally activate stress survival proteins such as heat shock protein 27 (Hsp27), peroxiredoxin 6 (PRDX6), and vascular endothelial growth factor C (VEGF-C) [18,26–30].

We observed previously that LEDGF/p75 overexpression in PCa cells did not protect against caspase-dependent apoptosis triggered by TRAIL (tumor necrosis factor related apoptosis inducing ligand), a well-characterized inducer of the death receptor apoptotic pathway [13]. TRAIL, staurosporine (STS), and other inducers of apoptosis lead to caspase-3 mediated cleavage of LEDGF/p75 into a prominent p65 fragment that lacks pro-survival activity and enhances cell death under stress conditions [22,23,30]. Furthermore, caspase-3 mediated cleavage of LEDGF/p52, the short alternative splice variant of LEDGF/p75, generates a p35 fragment that abrogates the transcriptional activity of LEDGF/p75 [30].

Because of its cleavage and inactivation during apoptosis, LEDGF/p75 may not act as a classical inhibitor of apoptosis but rather as an upstream protector of DNA and lysosomal integrity under an augmented state of cellular oxidative stress. Therefore, we focused the present study on investigating the ability of LEDGF/p75 to protect PCa cells against oxidative stress-induced necrosis, and contribute to the upregulation of endoplasmic reticulum protein of 57 kD (ERp57) in PCa. ERp57, also known as glucose regulated protein of 58 kD (GRP58) and protein disulfide isomerase family A member 3 (PDIA3), is a multi-functional thiol-oxidoreductase and a chaperone protein responsible for maintaining the appropriate folding of newly synthetized glycoproteins [31,32]. Our results indicate that LEDGF/p75 overexpression attenuates oxidative stress-induced necrotic cell death and contributes to ERp57 upregulation in the context of PCa.

## Materials and Methods

The studies involving human antibodies, cancer cell lines, and prostate tissues were performed under the approval of the Loma Linda University Institutional Review Board.

### Cell lines, Antibodies, and Reagents

The metastatic prostate cancer cell lines DU145 (Cat.# HTB-81) and PC3 (Cat.# CRL-1435), the K-ras transformed prostate epithelial cell line RWPE-2 (Cat.# CRL-11610), derived from the normal prostate cell line RWPE-1, and the osteosarcoma cell line U2OS (Cat.# HTB-96) were purchased from the American Type Culture Collection (ATCC). Cells were cultured as recommended by the suppliers in a humidified incubator with 5% CO_2_ at 37°C. The following antibodies were used: mouse monoclonals anti-β-actin (1:5000, Sigma-Aldrich) and anti-ERp57 (1: 200, Enzo Life Sciences); rabbit polyclonal anti-LEDGF/p75 (1:1000, Bethyl laboratories Inc); goat polyclonal anti-Lamin B antibody C-20 (1:1000. Santa Cruz Biotechnology); human autoantibody to topoisomerase I (1:100, Topo I), a kind gift from Dr. Eng M Tan (Scripps Research Institute, La Jolla, CA); anti-LEDGF/p75 rabbit polyclonal antibody Scripps-Ab5087(1:1000), also donated by Dr. Eng M. Tan; and horseradish peroxidase (HRP)-labeled secondary IgG antibodies (1:5000, ThermoFisher Scientific). Tert-butyl hydrogen peroxide (TBHP), an organic peroxide, was purchased from Sigma-Aldrich. STS and N-acetyl-Asp-Glu-Val-Asp-7-amino-4-methylcoumarin (Ac-DEVD-amc, fluorogenic caspase-3/7 substrate) were purchased from Axxora. The broad caspase inhibitor benzylocarbonyl-Val-Ala-Asp-fluoromethyl ketone (z-VAD-fmk) was purchased from Biomol International.

### Cell Death and Viability Assays

Cell death was induced by treatment with the cytotoxic agents TBHP (50, 75, 100, and 150 μM) or STS (4 μM) for up to 24 h. In some experiments cells were preincubated with 100 μM of z-VAD-fmk for 1 h prior to exposure to these agents. Cell morphology was visualized on an Olympus IX70 microscope equipped with Hoffmann Modulation Contrast (Olympus American) and a digital Spot Imaging System (Diagnostic Instruments). To determine cell viability, cells seeded in 96-well plates (3 x 10^4^ cells per well) were treated with TBHP or STS, washed with phosphate buffered saline (PBS), and fixed in 4% paraformaldehyde for 1 h at 4°C. Cells were then washed three times with distilled water, and Accustain Crystal Violet solution (Sigma-Aldrich) (1:4) was added to each well followed by incubation for 20 minutes at room temperature. Cells were washed with distilled water to remove excess dye and then dried at room temperature. Acetic acid (10% v/v) was added to each well for 10 minutes and absorbance was measured at 570 nanometers (nm) using a μQuant microplate reader (Bio-Tek Instruments).

DAPI (4',6-diamidino-2-phenylindole) staining was used to visualize condensed or fragmented chromatin in cells treated with TBHP or STS. Briefly, cells were seeded (3 x 10^4^) in 8-well Lab-Tek^®^ Permanox^®^ chamber slides (ThermoFisher Scientific), treated after 24 h with the different cytotoxic agents, washed with PBS, and then fixed and permeabilized with methanol/acetone solution (3:1 v/v) for 10 minutes at -20°C. The fixing solution was removed and the slides were incubated at room temperature for 5–10 minutes for drying. Coverslips were mounted using VectaShield mounting medium containing DAPI (Vector Laboratories). DAPI staining was visualized using and Olympus BX50 Fluorescence Microscope and images were obtained with a Spot Imaging System (Diagnostic Instruments).

Caspase activity assays were performed as described previously [30]. Briefly, cells were seeded in black, clear-bottomed 96-well plates (3 x 10^4^ cells per well). At the conclusion of treatment with TBHP or STS, cells were incubated with 50 μl of 3X caspase buffer [150 mM HEPES pH 7.4, 450 mM sodium chloride, 150 mM potassium chloride, 30 mM magnesium chloride, 1.2 mM ethylene glycol-bis(2-aminoethylether)-*N*,*N*,*N′*,*N′*-tetraacetic acid (EGTA), 30% sucrose, 10% CHAPS, and 1.5% NP-40], in the presence of 30 mM dithiothreitol (DTT), 3 mM phenylmethanesulphonylfluoride (PMSF), and 75 μM of the fluorogenic peptide substrate Ac-DEVD-amc (caspase-3/7) for 2 h at 37°C. This was followed by incubation of cells at room temperature for 12 h, and measurement of the absorbance at excitation of 360 nm and emission of 460 nm in a FLX800 Microplate Fluorescent Reader (Bio-tek Instruments). Fold activity was determined by normalizing to one the absorbance values for untreated, control cells.

### Measurement of Reactive Oxygen Species

The generation of reactive oxygen species (ROS) was assessed based on the intracellular oxidation of 2’,7’-dichlorodihydrofluorescein diacetate (DCFH-DA, Invitrogen) to form the fluorescent compound 2’,7’-dicholorofluorescein (DCF). Cells were seeded in a 6-well plate at a density of 3 x 10^4^ cells per well, cultured for 24 hours, and then treated with TBHP or STS for up to 12 h. DCFH-DA (0.5 μM) was then added to the cells, followed by incubation for 20 minutes at 37°C. Cells were washed with PBS and then resuspended in 0.5 mL of PBS. Fluorescence intensity was determined by flow cytometry using a FACScalibur cytometer (BD Biosciences).

### Quantitative Real Time PCR

Quantitative Real Time PCR (qPCR) was carried out as described previously [24]. Briefly, total RNA was extracted from cells using the RNeasy plus mini kit (Qiagen). RNA (0.5μg) was reverse trancribed into cDNA using iScript cDNA synthesis kit (BioRad). qPCR was performed on the MyiQ real-time PCR detection system with primers using iQ SYBR Green Supermix (BioRad) according to the manufacturer’s recommendations. Primer sequences for LEDGF/p75 and ERp57 were designed using the Primer3 software and commercially synthesized by Integrated DNA Technologies (IDT) (Table 1). Target mRNA values were normalized using glyceraldehyde 3-phosphate dehydrogenase (GAPDH) mRNA and data were expressed relative to normalized values of corresponding controls. Samples were analyzed in three independent experiments, each in triplicates.

**Table 1:** Nucleotide sequences of primers used for qPCR.

Gene	Forward sequence (5' to 3')	Reverse Sequence (5' to 3')
**LEDGF/p75**	TGCTTTTCCAGACATGGTTGT	CCCACAAACAGTGAAAAGACAG
**ERp57**	GGTGTGGACACTGCAAGAGA	AGCACCTGCTTCTTCACCAT
**GAPDH**	CGAGATCCCTCCAAAATCAA	TTCACACCCATGACGAACAT
**ERp57pr A**	GCCCACTCAGTCCTGACTTC	ACTCGTTACCGCCGAGTG
**ERp57pr B**	CAAGACGGCATCTCAGACAA	GCTTTGGCATTTTGTCCAAT
**ERp57pr C**	ACTAGGAGCAGTTGGGCAGA	CGTGTTAGCCAGGATGGTCT
**ERp57pr D**	CCTCCCCAGAATTTTCCACT	CTGTAATCCTTGGTGCAGCTC
**ERp57pr E**	CCTCTGGGCATGTGAAATCT	CCAGCAGTAAGCCATTAGGG

### Generation of PCa cells with stable overexpression or depletion of LEDGF/p75

LEDGF/p75 cDNA was previously cloned in our laboratory into the mammalian expression plasmid pcDNA3.1 (Invitrogen) [22]. Briefly, both pcDNA3.1 empty vector and pcDNA3.1-*ledgf/p75* plasmids were transfected into RWPE-2 and PC3 cells using the Fugene 6 (Roche) method. Forty-eight hours post-transfection, cells were trypsinized and seeded into 6-well plates. Selection of stable transfectants was achieved by adding G418 (330μg/ml) (ThermoFisher Scientific) to the cell cultures. Surviving colonies were expanded and the expression of LEDGF/p75 in cells stably transfected with empty vector or pcDNA3.1-*ledgf/p75* was confirmed by qPCR and immunoblotting.

PC3 cells with stable overexpression or depletion of LEDGFp75 using viral vectors were generous gifts from Professors Zeger Debyser and Rik Gijsbers (Katholieke Universiteit Leuven, Belgium). Overexpressing PC3 cells were generated by transducing them with retroviral vectors encoding full-length LEDGF/p75 cDNA as described previously [24,33] PC3 cells with stable depletion of LEDGFp75 were generated using intensified lentiviral vector-based RNA interference as described previously [34]. Briefly, short hairpin (sh) RNA was used to stably knockdown LEDGFp75 while shSCR served as the non-interfering shRNA control. Transduced cells were selected with zeocin (200 μg/ml) and LEDGF/p75 overexpression or depletion in selected clones was assessed by qPCR and immunoblotting.

### RNA interference-mediated knockdown of LEDGF/p75 in PCa cells

Transient knockdown of LEDGF/p75 in PCa cells was achieved by delivering specific short inhibitory RNAs (siRNAs) into cells using the Oligofectamine method (Invitrogen, Life Technologies), according to manufacturer’s instructions. Briefly, LEDGF/p75 siRNA (siLEDGF/p75) and a scrambled siRNA duplex (siSD, negative control) were designed as described previously [24] and synthesized by IDT. The siLEDGF/p75 sequence corresponded to nucleotides 1340–1360 (5′- AGACAGCAUGAGGAAGCGAdTdT-3′) with respect to the first nucleotide of the start codon of the LEDGF/p75 open reading frame. This sequence corresponds to a region in the C-terminus of LEDGF/p75 that is not shared by its short alternative splice variant LEDGF/p52. The sequence for siSD was 5′- GCGCGCUUUGUAGGAUUCGdTdT-3′. Cells were transfected with 40 nM siRNAs and grown for 72 hours before analysis.

### Docetaxel-Resistant PCa Cells

Docetaxel (DTX)-resistant PC3 (PC3-DR) and DU145 (DU145-DR) cell lines were developed by culturing PC3 and DU145 cells in the presence of DTX in a dose-escalation manner [35]. Cells that survived the initial culture in 1 nM DTX were passaged 4 times prior to increasing the concentration of DTX to 5.5 nM and subsequently to 11 nM. Resistant cells were maintained continuously in 11 nM DTX.

### Immunoblotting Analysis

Immunoblotting was carried out essentially as described previously [24]. Briefly, proteins in whole-cell lysates from PCa cells were separated by SDS-PAGE (NuPAGE 4–12%, ThermoFisher Scientific) followed by transfer to polyvinyl difluoride (PVDF) membranes (Millipore). Membranes were blocked with 5% dry milk solution prepared in TBS-T buffer (20 mM Tris-HCl, pH 7.6, 140 mM NaCl, 0.1% Tween 20) and probed with primary antibodies. After several washes with TBS-T, membranes were incubated with appropriate horseradish peroxidase (HRP)-conjugated secondary antibodies and washed again several times with TBS-T. Protein bands were detected by enhanced chemiluminescence (ThermoFisher Scientific Pierce).

### Kinetworks™ Stress/Heat Shock Protein Screen

The Kinetworks™ Stress/Heat Shock Protein Screen (Kinexus Bioinformatics Corporation) was used to quantify and compare the expression levels of 25 different stress and heat shock proteins in immunoblots of whole cell lysates from RWPE-2 cells stably overexpressing LEDGF/p75 and control cells stably transfected with empty pcDNA vector. This platform was an antibody-based array service for the simultaneous screening of multiple stress proteins in cell lysates that was commercially available at the time we initiated this study. Cell lysates were probed by immunoblotting against a stress/heat shock protein antibody panel [36]. Immunoblotting procedures and quantification of individual stress protein immunoreactivity were performed by Kinexus.

### Luciferase-based Transcription Reporter Assays

ERp57 promoter (*ERp57pr*) luciferase-based transcription reporter assays were performed as described previously [30]. Briefly, RWPE-2, PC3, and DU145 cells were co-transfected with the expression vector encoding LEDGF/p75 (pcDNA3.1-*ledgf/p75*) or empty vector (pcDNA3.1), and the reporter vector (pLightSwitch empty vector, or pLightSwitch-*ERp57pr*) (Switchgear Genomics/Active Motif). At 48 hours post-transfection, cells were lysed and luciferase assays were performed using the LightSwitch Luciferase Assay Reagent (Switchgear Genomics/Active Motif). U2OS cells were transfected with a different LEDGF/p75 expression vector, pCruzHA-*ledgf/p75*, or empty pCruzHA, and luciferase assay in these cells was performed using the Luciferase Assay System from Promega. Relative light units were obtained in a MicroLumatPlus Lb 96V luminometer (Berthold Tech), and luciferase values were normalized to protein concentration of lysates from cells co-transfected with empty vectors and pLightSwitch-*ERp57pr*. Student’s *t*-test analysis was performed using Microsoft Excel. Experiments were repeated at least three times in triplicates.

### Chromatin Immunoprecipitation Assays

These assays were conducted essentially as described previously [37]. Briefly, PC3 and U2OS cells were fixed in 1% formaldehyde for 10 minutes and subjected to chromatin immunoprecipitation (ChIP) assay using the ChIP-IT Express Enzymatic kit (Active Motif). Anti-LEDGF/p75 antibodies (A300-848A, Bethyl) was used to immunoprecipitate protein-chromatin complexes. Immunoprecipitated chromatin was then enzymatically digested. PCR was performed using specific primers to amplify the ERp57 promoter (Table 1). Both non-specific immunoglobulin G (IgG) antibody and input chromatin were used as controls.

### Immunohistochemical (IHC) Analysis of Prostate Cancer Tissue Microarrays

Human PCa tissue microarrays (TMAs), commercially available from Novus Biologicals and US Biomax Inc. were used for IHC analysis of LEDGF/p75 and ERp57. Three different PCa TMAs (two from US Biomax Inc. and one from Novus Biologicals) were used to increase the sample size. Briefly, we acquired from US Biomax the PR807 and PR807a TMAs, each containing single cores of 3 disease-free normal tissue cases, 7 normal adjacent tissue cases, and 50 PCa tissue cases. We also used the IMH-303 TMA (Novus Biologicals), containing 40 PCa tissue cores and 9 matched normal adjacent tissues. The manufacturers of these TMAs provided only limited basic clinicopathological information (age, sex, tumor stage in some cases) corresponding to the tissue cores, with no patient identifiers. No information was available on patient race or ethnicity, treatment type, institutions that collected the tissues, follow- up routines, and tissue handling techniques. The limited patient follow-up data associated with the TMAs prevented us from performing a Kaplan-Meier survival analysis and other clinical correlation analyses.

TMAs were stained using a Biogenic i6000 auto-stainer (Biogenex Corporation) as described previously [11]. Briefly, paraffin embedded tissue sections in the TMA slides were deparaffinized and the slides were subjected to antigen retrieval. Endogenous peroxidase activity was quenched by treatment with 3% hydrogen peroxide in 10% methanol, and Power Block^©^ universal blocking reagent (Biogenex Corp.) was used to block non-specific protein binding. IHC staining of LEDGF/p75 was done using the Scripps-Ab5087 rabbit polyclonal antibody, which is specific for LEDGF/p75 and does not react with the short LEDGF/p52 splice variant [11]. IHC staining of ERp57 was done using the mouse monoclonal anti-ERp57 (Enzo Life Sciences). TMA slides were incubated overnight with primary antibodies, washed and then incubated with Multi-link^©^ biotinylated secondary antibody (Biogenex Corp.), followed by incubation with streptavidin-coupled peroxidase supersensitive Label^©^ (Biogenex Corp.). Immunostaining was detected by peroxidase activation of the 3-amino-9-ethycarbazole (AEC) chromagen (Biocare Medical). TMAs were counterstained lightly with hematoxylin (Sigma) and mounted with permount (ThermoFisher Scientific). For the negative control sample, the primary antibody was omitted and substituted with rabbit or mouse pre-immune serum. Tissue sections were examined under an Olympus BX50 microscope, and images were acquired using a digital Spot RT3^TM^ camera (Diagnostic Instruments).

Immunostained TMAs were scored blindly for LEDGF/p75 immunoreactivity by a board certified pathologist (HR). A 4-tier scoring system (0 = negative, 1 = weak, 2 = moderate, 3 = strong) was used to evaluate staining intensity. Tissues with scores of 0–1 were considered to have low intensity staining, whereas tissues with scores of 2–3 were considered to have high intensity staining. Tissue specimens that showed poor quality were excluded from the analyses. These studies were performed under approval by the Institutional Review Board.

Statistical analysis of IHC data and their relationship to patients’ clinical outcomes was done using the SAS software package (version 9.2; SAS institute). For ease of statistical analysis, tissue specimens were grouped into two categories based on their scores. ‘Low’ staining was determined as pooled staining intensity scores of 0 and 1 while ‘high’ staining had pooled scores of 2 and 3. Correlation between expression levels of LEDGF/p75 and ERp57 in tumor and control (disease-free normal + normal adjacent) tissues was determined using Chi-square test. Probability values below 0.05 were considered significant.

## Results

### LEDGF/p75 overexpression attenuates oxidative stress-induced necrosis but not staurosporine-induced apoptosis in PCa cells

**Fig 1:**
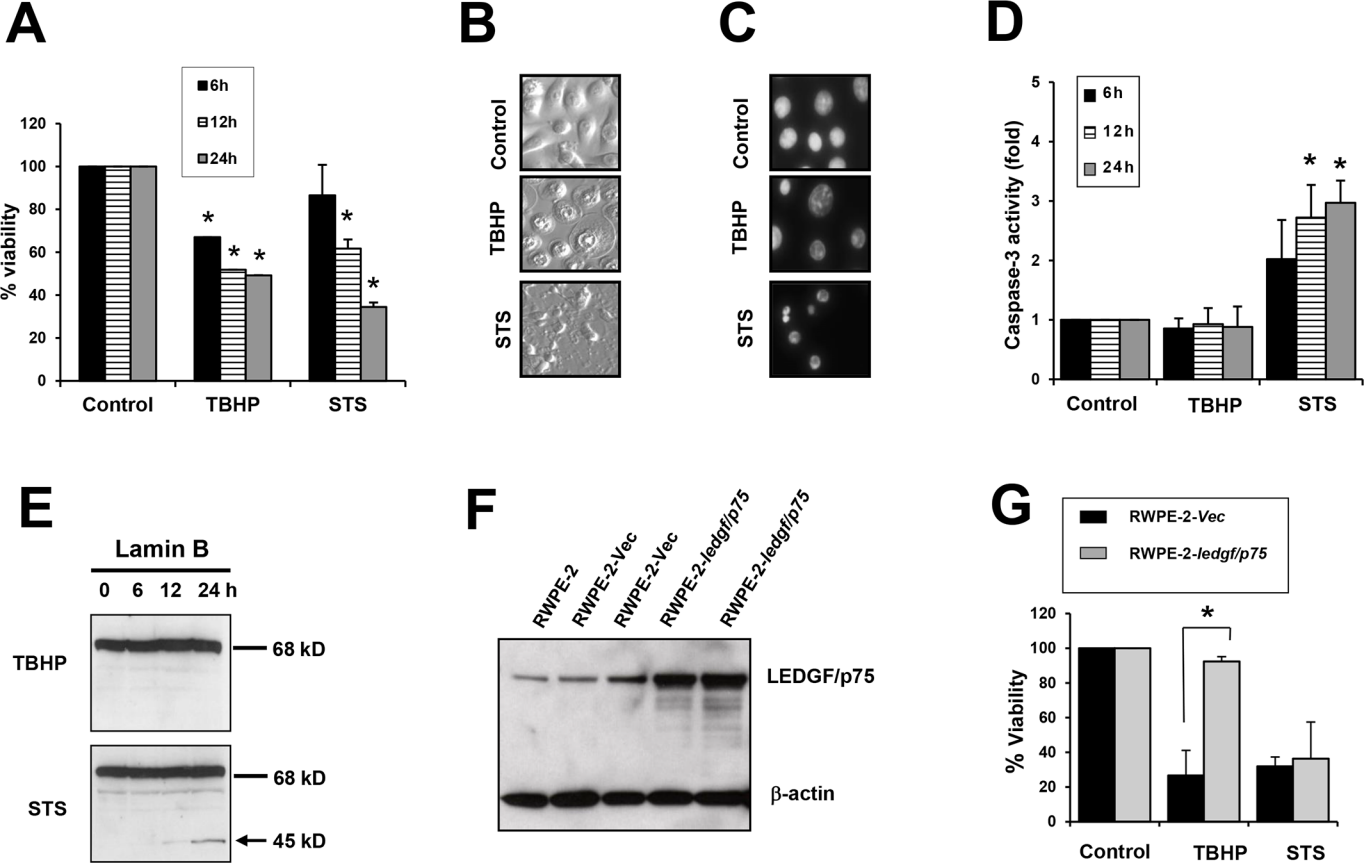
Overexpression of LEDGF/p75 in RWPE-2 cells attenuates TBHP-induced necrosis but not STS-induced apoptosis. A. RWPE-2 cells were treated with 100 μM TBHP or 4 μM STS to induce necrosis or apoptosis, respectively. Cell viability was assessed by Crystal Violet assay. B. Changes in cellular morphology associated with necrosis or apoptosis in RWPE-2 cells treated with TBHP or STS, respectively, for 24 hours. C. Nuclear morphology of RWPE-2 cells (treated as in panel B) visualized by DAPI staining. D. Caspase 3 activity was measured after treatment with TBHP or STS. E. Cleavage of Lamin B into its signature apoptotic 45 kD fragment was detected by immunoblotting in RWPE-2 cells treated with STS but not in TBHP-treated cells. Lines indicate bands corresponding to intact proteins and the arrow points to the cleavage fragment. F. Immunoblot showing stable overexpression of LEDGF/p75 in RWPE-2–*ledgf/p75* clones as compared to RWPE-2 *Vec* (empty pcDNA3.1 vector) clones and untransfected RWPE-2 cells. G. Crystal violet viability assay showing that overexpression of LEDGF/p75 in RWPE-2 cells promotes resistance to cell death induced by TBHP but not STS. Each graph represents the average of at least 3 independent experiments performed in triplicates (*P<0.*05)*. *P* values were determined in comparison to control using the Student’s *t*-test. Data represent the average of at least independent experiments.

TBHP, a more stable homologue of hydrogen peroxide that is widely used in models of oxidative stress-induced necrosis [38–40], and the apoptosis inducer STS were exposed to PCa cells to activate necrotic and apoptotic cell death, respectively. Both agents reduced cell viability in the RWPE-2 prostate cell line (Fig 1A). However, while STS-treated cells presented the classical apoptotic hallmarks characterized by blebbing and chromatin condensation and fragmentation, TBHP-treated cells exhibited the typical necrotic morphology characterized by cytoplasmic swelling and condensed nuclei without chromatin fragmentation (Fig 1B and 1C). Treatment with STS led to a time-dependent increase in caspase-3 activity, consistent with apoptosis induction, whereas treatment with TBHP did not lead to caspase-3 activation (Fig 1D). Lamin B, a classical caspase-3 substrate, was cleaved into its signature apoptotic fragment of 45 kD during STS-induced apoptosis but not during TBHP-induced cell death (Fig 1E), consistent with our previous observation that this protein is not cleaved during necrotic cell death [41]. Taken together, these results were consistent with available evidence that TBHP is a strong trigger of oxidative stress-induced necrotic cell death [38–40].

To determine if LEDGF/p75 antagonizes TBHP-induced necrosis, we generated RWPE-2 cells stably overexpressing this protein (Fig 1F). These cells displayed a significant attenuation of TBHP-induced necrotic cell death compared to cells stably transfected with the empty pcDNA3.1 vector (Fig 1G). As expected, LEDGF/p75 overexpression did not attenuate STS-induced apoptosis (Fig 1G), most likely due to its cleavage by caspases [22,23,30].

**Fig 2:**
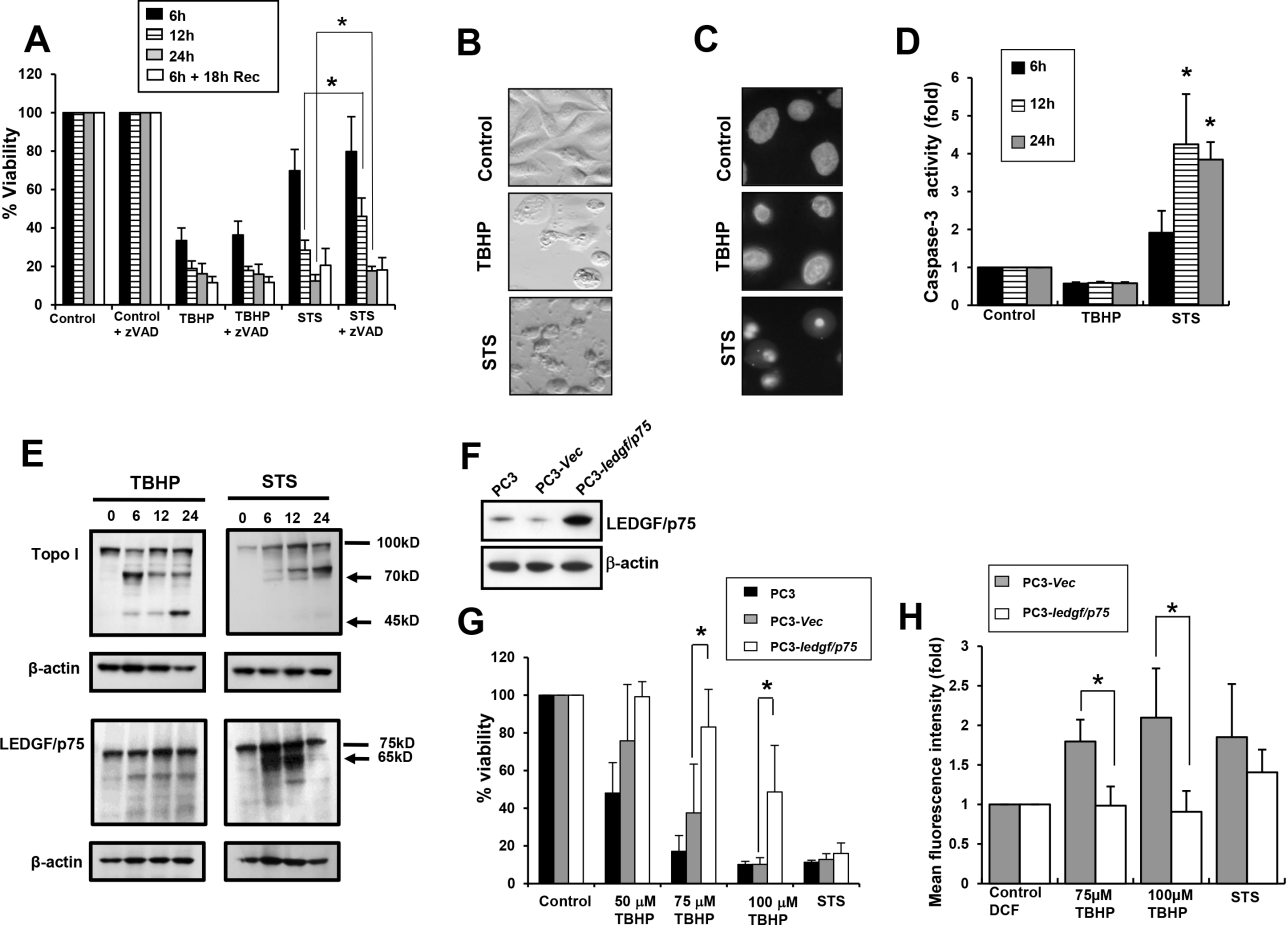
Overexpression of LEDGF/p75 in PC3 cells attenuates TBHP-induced necrosis but not STS-induced apoptosis. A. PC3 cells were treated with 100 μM TBHP or 4 μM STS to induce necrotic or apoptotic cell death, respectively, in the presence and absence of the broad caspase inhibitor zVAD-fmk. Cell viability was assessed at 6, 12, 24 h post-treatment and, at 6 h treatment plus 18 h recovery. B. Changes in cellular morphology associated with necrosis or apoptosis in PC3 cells treated with TBHP and STS, respectively, for 24 h. C. Nuclear morphology of PC3 cells visualized by DAPI staining. D. Caspase 3 activity was measured after treatment with TBHP or STS. E. Cleavage of Topo I into its signature apoptotic (70 kD) and necrotic (70 kD and 45 kD) fragments was detected by immunoblotting in PC3 cells treated with STS or TBHP, respectively (upper panel). Cleavage of LEDGF/p75 into its signature apoptotic fragment (65 kD) was detected in cells treated with STS but not in cells treated with TBHP (lower panel). β-actin was used as loading control. Lines indicate bands corresponding to intact proteins and arrows point to cleavage fragments. F. Immunoblot showing stable overexpression of LEDGF/p75 in PC3–*ledgf/p75* clones as compared to PC3-*Vec* (empty pcDNA3.1 vector) or untransfected parental PC3 cells. G. Crystal violet viability assay showing that overexpression of LEDGF/p75 in PC3 cells promotes resistance to cell death induced by 75 and 100 μM TBHP but not STS. H. DCFH-DA oxidation flow cytometric analysis to measure the ability of LEDGF/p75 to reduce ROS induced by TBHP or STS in PC3–*ledgf/p75* cells as compared to cells transfected with empty vector (PC3-*Vec*), in the presence or absence of TBHP or STS. Each graph represents the average of at least 3 independent experiments performed in triplicates (*P<0.*05)*. *P* values were determined in comparison to control using Student’s *t*-test.

We reproduced and expanded these findings in the bone metastatic PCa cells, PC3. Similar to our observations in RWPE-2 cells, treatment of PC3 cells with either STS or TBHP reduced cell viability in a time dependent manner (Fig 2A). As expected, zVAD-fmk, a broad inhibitor of caspases attenuated STS-induced apoptosis but had no inhibitory effects on TBHP-induced necrosis (Fig 2A). Although zVAD-fmk significantly attenuated STS-mediated apoptosis, it did not completely inhibit cell death as time progressed (Fig 2A), consistent with previous observations that STS induces caspase-independent cell death or necroptosis under caspase-compromised conditions in certain cell lines [42,43]. Consistent with our observations in RWPE-2 cells, STS-treated PC3 cells exhibited the characteristic blebbing and extensive chromatin condensation and fragmentation associated with apoptosis, whereas TBHP-treated cells exhibited cytoplasmic swelling without nuclear fragmentation (Fig 2B and 2C). As expected, treatment with STS, but not with TBHP, led to caspase-3 activation (Fig 2D).

To further strengthen these findings we examined by immunoblotting the cleavage pattern of Topoisomerase I (Topo I) and LEDGF/p75 during treatment of PC3 cells with either STS or TBHP. We have previously demonstrated in several studies that Topo I is cleaved by caspases during apoptosis into a fragment of 70 kD, and by cathepsins during necrosis or necroptosis into fragments of 70 and 45 kD [41,44,45]. This led us to propose that cleavage of Topo I into a 45 kD fragment is a marker of necrotic cell death [41,44,45]. In agreement with our previous observations, Topo I was cleaved into fragments of 70 and 45 kD in cells treated with TBHP, consistent with induction of necrosis, and into a single fragment of 70 kD in cells treated with STS, consistent with induction of apoptosis (Fig 2E, top panels). Similar to the results obtained with RWPE-2 cells, these results in PC3 cells were consistent with TBHP triggering oxidative stress-induced necrosis.

As expected, LEDGF/p75 was robustly cleaved into its signature, caspase-3-generated fragment of 65 kD in STS-treated cells (Fig 2E, bottom panels). We showed previously that this p65 fragment not only lack stress survival activity but accelerates cell death in HepG2 liver cancer cells [22]. The band corresponding to this fragment was very faint after 24 h, likely due to the extensive degradation of late apoptotic/secondary necrotic cells at this time point. The residual 75 kD band corresponding to LEDGF/p75 that still appeared at 24 h is derived from cells that survived STS-induced apoptosis. This pro-apoptotic p65 fragment was not present in the TBHP-treated cells (Fig 2E). Instead, we observed that LEDGF/p75 stayed relatively intact in these cells, showing a weaker band of approximately 60 kD that is likely related to a necrosis-related minor degradation product observed previously [45].

We then sought to determine if LEDGF/p75 would antagonize TBHP-induced necrosis in PC3 cells. To accomplish this, we generated PC3 cells stably overexpressing LEDGF/p75 (Fig 2F), and examined their ability to protect cells against TBHP-induced necrosis. Similar to our findings in RWPE-2 cells, PC3 cells overexpressing LEDGF/p75 also displayed significant attenuation of TBHP-induced necrosis when compared to cells stably transfected with the empty vector (Fig 2G). This protection was observed at TBHP concentrations of 75 and 100 μM. It was also observed at 50 μM but it was not statistically significant. We did not observe protection at concentrations above 150 μM because these were too toxic to the cells (data not shown). As predicted, LEDGF/p75 overexpression did not attenuate STS-induced apoptosis in these cells (Fig 2G), most likely due to its conversion to the pro-apoptotic p65 fragment. These results were confirmed by morphological examination of cells overexpressing LEDGF/p75 or transfected with empty vector, in the presence and absence of the drugs (data not shown). These findings were also reproduced in U2OS cells overexpressing LEDGF/p75 (data not shown).

To determine whether LEDGF/p75-induced protection against oxidative stress-induced necrosis is associated with decreased ROS generation, a DCFH-DA oxidation analysis was performed with PC3 cells stably overexpressing this protein or transfected with empty vector, in the presence or absence of TBHP or STS. We observed that PC3 cells overexpressing LEDGF/p75 and treated with TBHP showed significantly decreased mean DCF fluorescence intensity compared to control cells transfected with empty vector (Fig 2H). However, there was no significant difference in the mean DCF fluorescence intensity between empty vector transfected and LEDGF/p75-overexpressing PC3 cells treated with STS. These results indicated that LEDGF/p75 overexpression reduces endogenous ROS levels generated by TBHP, consistent with its role as a transcription co-activator that promotes cellular resistance against oxidative stress-induced cell death by upregulating stress response proteins [7,8,17,24–30,37].

### LEDGF/p75 overexpression leads to ERp57 upregulation in PCa cells

**Fig 3:**
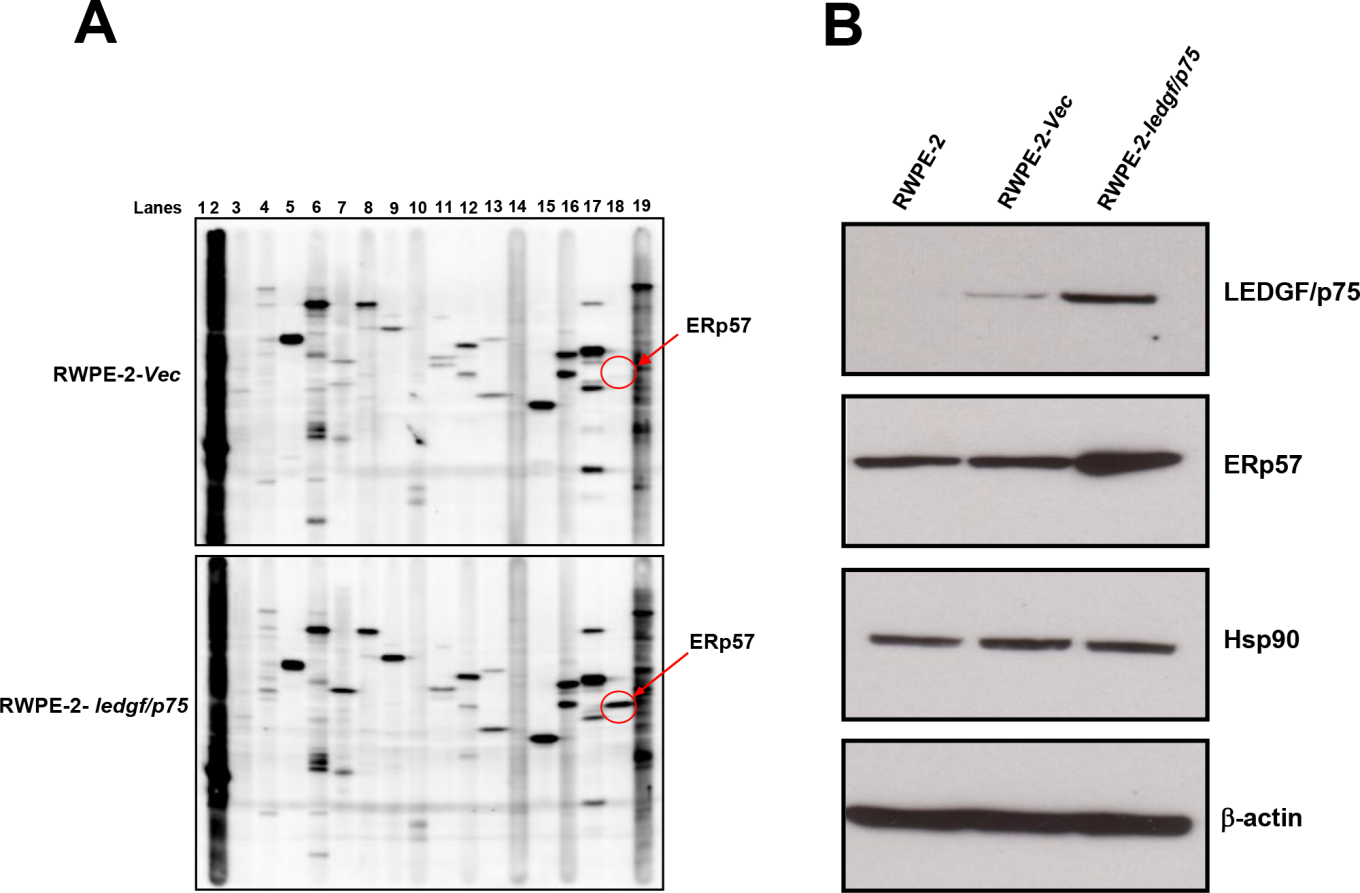
Multi-screen immunoblotting analysis to identify candidate stress proteins upregulated by LEDGF/p75 in RWPE-2 cells. A. Lysates from cells stably overexpressing LEDGF/p75 (RWPE-2–*ledgf/p75*) and RWPE-2 cells transfected with empty pcDNA3.1 vector (RWPE-2-*Vec*) were individually analyzed by immunoblotting using the Kinetworks™ KHSP-1.0 screen platform. Validation was performed using commercial antibodies and changes in protein expression were determined in LEDGF/p75-overexpressing RWPE-2 cells in comparison to cells transfected with empty vector. B. Untransfected RWPE-2 cells, cells transfected with empty vector, and cells stably overexpressing LEDGF/p75 were tested in-house for additional validation of upregulation of ERp57 and Hsp90. β-actin was used as loading control.

To identify additional proteins that are upregulated following LEDGF/p75 overexpression in PCa cells we performed a stress/heat shock protein profiling. Lysates from RWPE-2 cells stably overexpressing LEDGF/p75, as well as from empty vector transfected cells, were subjected to an immunoblotting-based stress protein profiling that analyzed quantitatively the expression of 25 known heat shock and stress related proteins using validated commercial antibodies (Kinetworks™ KHSP-1.0 screen). Proteins profiled in this screen are listed in Table 2 and representative blots are shown in Fig 3A. The empty vector transfected cells were used to determine the basal expression level of these proteins. The data is presented as fold increase or decrease in protein expression in the LEDGF/p75-overexpressing cells compared to empty vector controls. Of the 25 proteins analyzed, only 3 showed upregulation above 2 fold (Table 2). ERp57 showed the highest upregulation with a 33.55 fold increase, followed by Hsp90α with a 4.54 fold increase, and Hop-p60 with a 3.17 fold increase.

**Table 2:** Changes in stress protein expression in response to stable overexpression of LEDGF/p75 in RWPE-2 cells as determined by multi-screen immunoblotting.

Lane No.	Band	Full name of protein recognized by screening antibody	Abbreviation	Fold change
2	1	Heme Oxygenase 2	HO-2	NC
3	1	Hsp70/Hsc70 interacting protein	Hip	NC
4	1	APG-1 heat shock protein	APG1	NC
5	1	ER protein 72 kDa	ERp72	NC
6	1	Heat shock 70 kD protein 4	APG2	NC
7	1	Stress inducible protein 1	STI1/Hop-p60	↑ 3.17
7	2	Eukaryotic translation initiation factor 2α	eIF2α	↑ 1.41
8	1	Heat shock 105 kDA protein	Hsp105	NC
9	1	Heat shock 90 kDA protein 1, alpha	Hsp90α	↑ 4.54
9	NB	DnaJ (Hsp40) homolog, subfamily B, member 1	Hsp40	NE
10	1	Heat shock 25 kDA protein 1	Hsp25	↑ 1.59
11	1	Glucose regulated protein 94	Grp94	NC
11	2	Human RAD23 homolog B	hHR23B	↑ 1.58
12	1	Glucose regulated protein 75	Grp75	NC
12	2	Heat shock 60 kDA protein 1 (chaperonin)	Hsp60	↓ 1.79
13	2	Heat shock transcription factor 4	HSF4	NC
14	NB	Glucose regulated protein 78	Grp78	NE
15	1	Heat shock protein 47	Hsp47	NC
15	NB	Cyclo-oxygenase 2	COX-2	NE
16	1	Heat shock 70 kDA protein 1	Hsp70	NC
16	2	Heat shock 60 kDa protein 1 (chaperonin)	GRoEL	NC
17	2	Heat shock 70 protein 8	Hsc70	NC
18	1	ER protein 57 kDa	ERp57	↑ 33.55
18	NB	Hsp70 binding protein 1	HspBP1	NE
19	NB	Heme Oxygenase 1	HO-1	NE

Numbers represent fold changes in protein expression levels in RWPE-2 cells stably overexpressing LEDGF/p75 compared to cells stably transfected with empty pcDNA vector.

↑ = Upregulation, ↓ = Downregulation, NC = No change in expression, NB = No band detected

**Fig 4:**
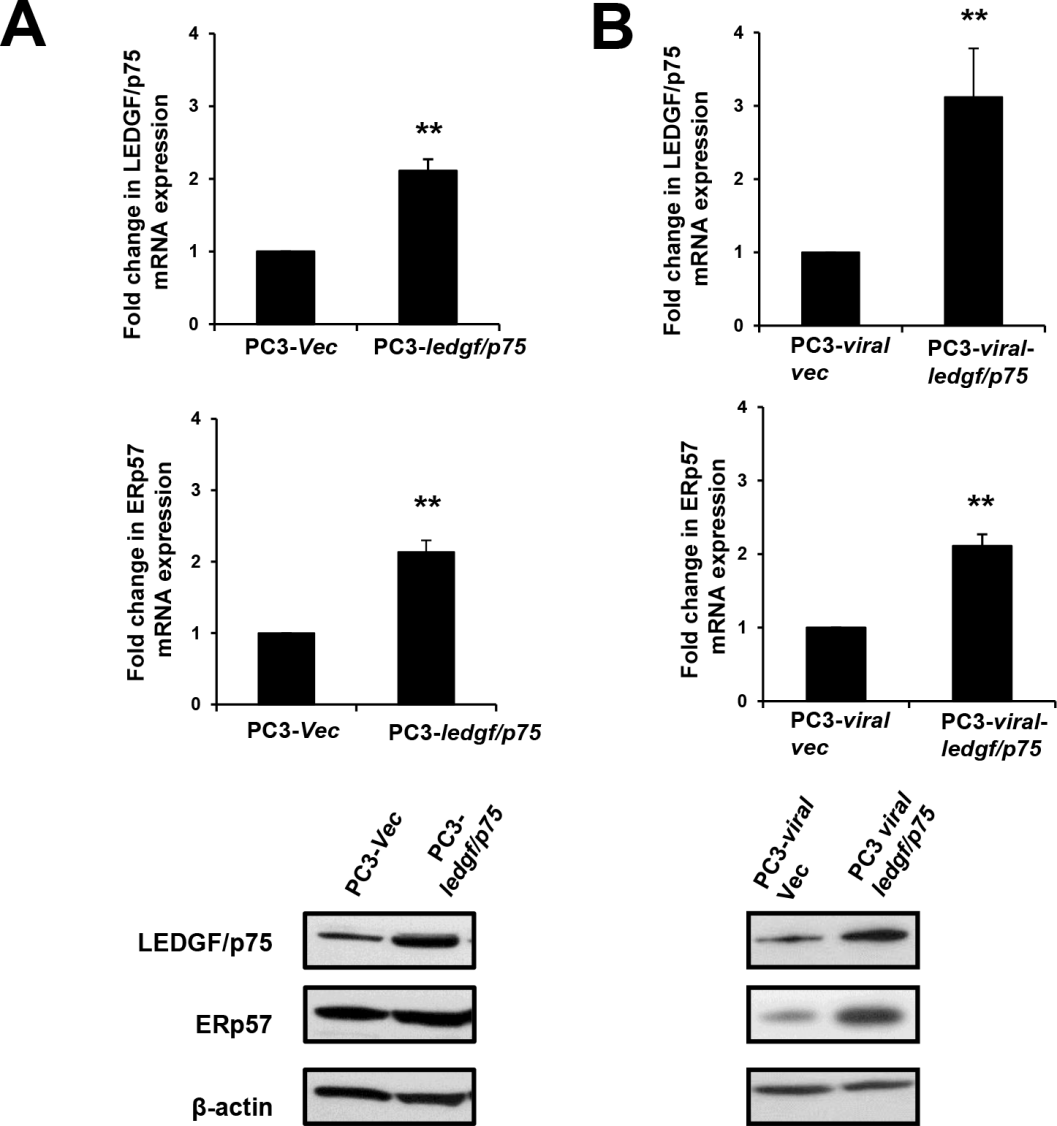
Upregulation of ERp57 expression levels in PC3 cells stably overexpressing LEDGF/p75. LEDGF/p75 and ERp57 transcript and protein levels were assessed by qPCR and immunoblotting, respectively. PC3 cells stably overexpressing LEDGF/p75 exhibited upregulation of ERp57 transcript and protein levels in PC3-*ledgf/p75* clones transfected with pcDNA3.1 plasmid (A) and in PC3-*viral*-*ledgf/p75* clones transfected with lentiviral vector (B). β-actin was used as loading control. Each graph represents the average of at least 3 independent experiments performed in triplicates (**P <0.01). *P* values were determined in comparison to cells transfected with empty vector as controls using the Student’s *t*-test.

To confirm these results, we focused on the two proteins with the highest fold upregulation and examined them with our own immunoblot analysis. We observed upregulation of ERp57 in RWPE-2 cells overexpressing LEDGF/p75 when compared to the parental cells as well as the empty vector transfected cells (Fig 3B). However, the expression levels of Hsp90 did not appear to change in the LEDGF/p75-overexpressing cells (Fig 3B). These results suggested that overexpression of LEDGF/p75 in RWPE-2 cells led to upregulation of ERp57. To determine if we could reproduce these results in other PCa cells, we utilized two different PC3 cell lines stably overexpressing LEDGF/p75, one using pcDNA3.1 plasmid and the other using a lentiviral vector. Consistent with the results obtained with RWPE-2 cells, these two clones displayed significantly increased transcript and protein expression of both LEDGF/p75 and ERp57 (Fig 4A and 4B).

**Fig 5:**
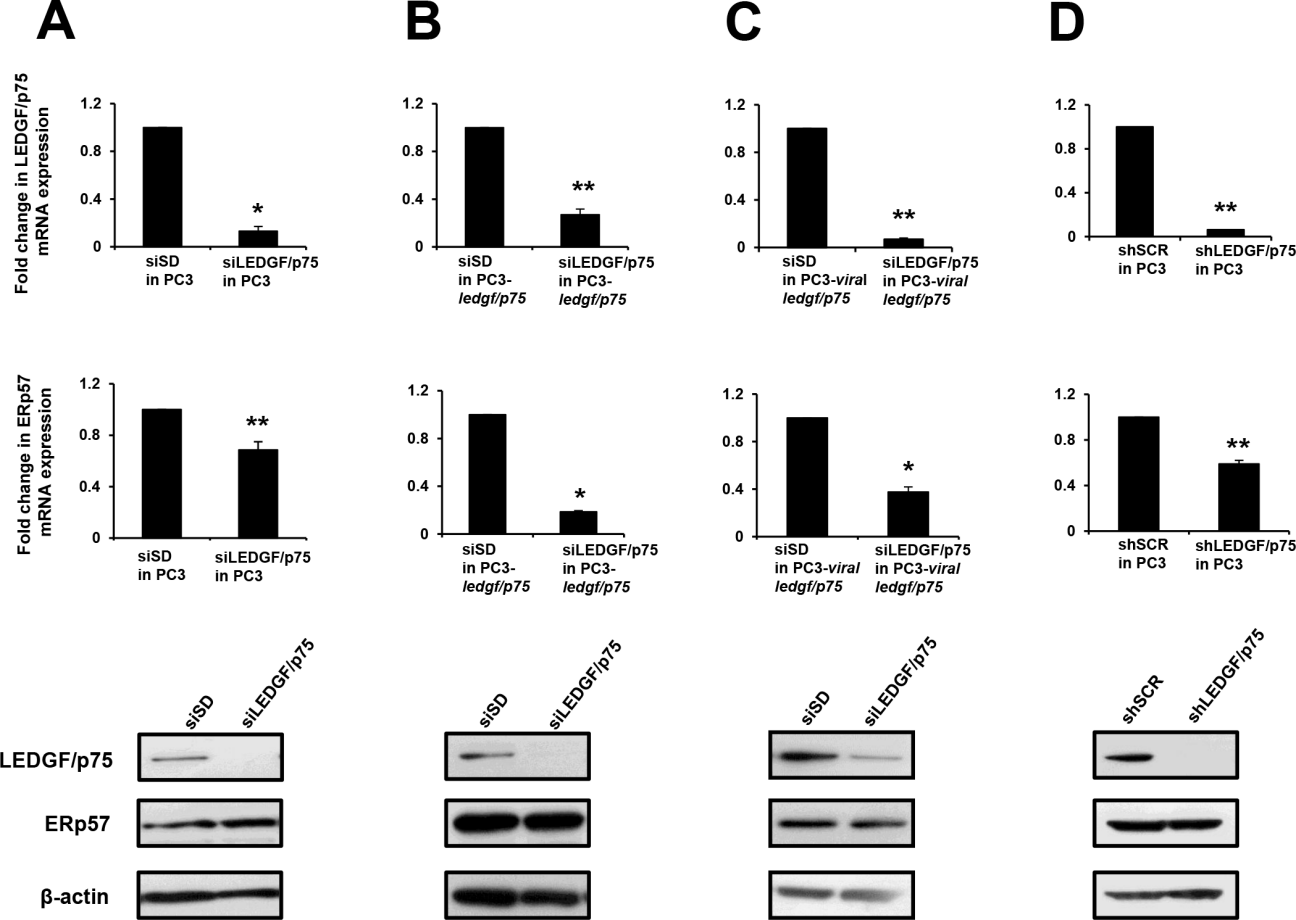
Effects of transient and stable knockdown of LEDGF/p75 on ERp57 expression levels in PC3 cells. LEDGF/p75 and ERp57 transcript and protein levels were assessed by qPCR and immunoblotting, respectively. A. Untransfected, parental PC3 cells with and without siRNA-induced transient depletion of LEDGF/p75; B. PC3-*ledgf/p75* clones overexpressing LEDGF/p75 with and without siRNA-induced transient depletion of LEDGF/p75; C. PC3-*viral-ledgf/p75* clones overexpressing LEDGF/p75 with and without siRNA-induced transient depletion of LEDGF/p75; D. PC3 cells with shRNA induced stable depletion of LEDGF/p75. Each graph represents the average of at least 3 independent experiments performed in triplicates (*P<0.*05*, **P <0.01). *P* values were determined in comparison to cells transfected with non-specific, scrambled control siRNAs (siSD or shSCR) using the Student’s *t*-test.

Next we investigated if LEDGF/p75 knockdown led to ERp57 downregulation. We used siRNAs to transiently deplete LEDGF/p75 in parental PC3 cells (Fig 5A) and in PC3 clones stably overexpressing this protein (Fig 5B and 5C). All the three LEDGF/p75-depleted cell lines exhibited significant downregulation of ERp57 transcript, suggesting a role for LEDGF/p75 in regulating ERp57 transcript expression (Fig 5A–5C). Surprisingly, no reduction in ERp57 protein expression was observed in these cell lines. Similar results were obtained when we used a PC3 cell line with shRNA-induced stable knockdown of LEDGF/p75 (Fig 5D).

**Fig 6:**
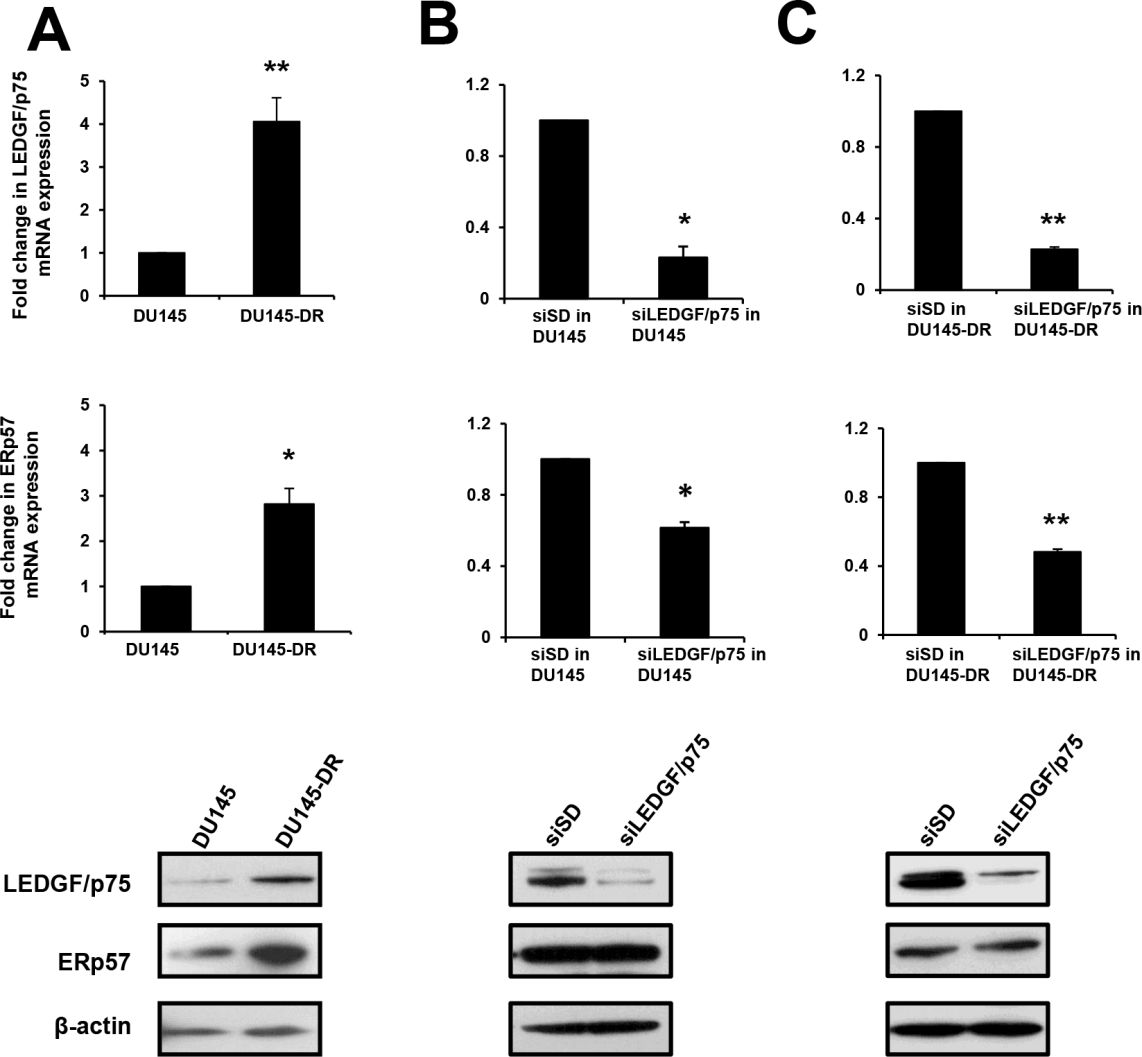
Effects of transient depletion of LEDGF/p75 on ERp57 expression levels in DU145 cells. A. LEDGF/p75 and ERp57 transcript and protein levels were assessed by qPCR and immunoblotting, respectively. A. Transcript and protein expression levels of LEDGF/p75 and ERp57 in DU145-DR cells, selected for their resistance to DTX, compared to parental DU145 cells. B. Parental DU145 cells with and without siRNA induced transient depletion of LEDGF/p75. C. DU145-DR cells with and without siRNA induced transient depletion of LEDGF/p75. Each graph represents the average of at least 3 independent experiments performed in triplicates (*P<0.*05*, **P <0.01). *P* values were determined in comparison to cells transfected with non-specific, scrambled control siRNAs (siSD) using the Student’s *t*-test.

In order to reproduce these findings in another PCa cell line in the context of chemoresistance, we explored the association between the expression of LEDGF/p75 and ERp57 in the DTX-resistant PCa cell line DU145-DR. We previously documented that DU145-DR cells express elevated levels of LEDGF/p75 compared to their parental cell line DU145, and that these elevated levels led to upregulation of the stress protein cytoglobin [24]. Quantitative PCR and immunoblotting analysis revealed significant upregulation of ERp57 transcript and protein in the DU145-DR cells (Fig 6A). Consistent with our results in PC3 cells, siRNA-induced transient knockdown of LEDGF/p75 in the parental DU145 and resistant DU145-DR cells reduced ERp57 transcript expression, as compared to the scrambled siRNA control, but did not cause dowregulation of ERp57 protein (Fig 6B and 6C). Taken together, all these results indicate that overexpression of LEDGF/p75 in PCa cells is associated with increased expression of ERp57 transcript and protein, whereas its depletion leads to decreased expression of the ERp57 transcript but not the protein.

### LEDGF/p75 binds to and transactivates the ERp57 promoter

**Fig 7:**
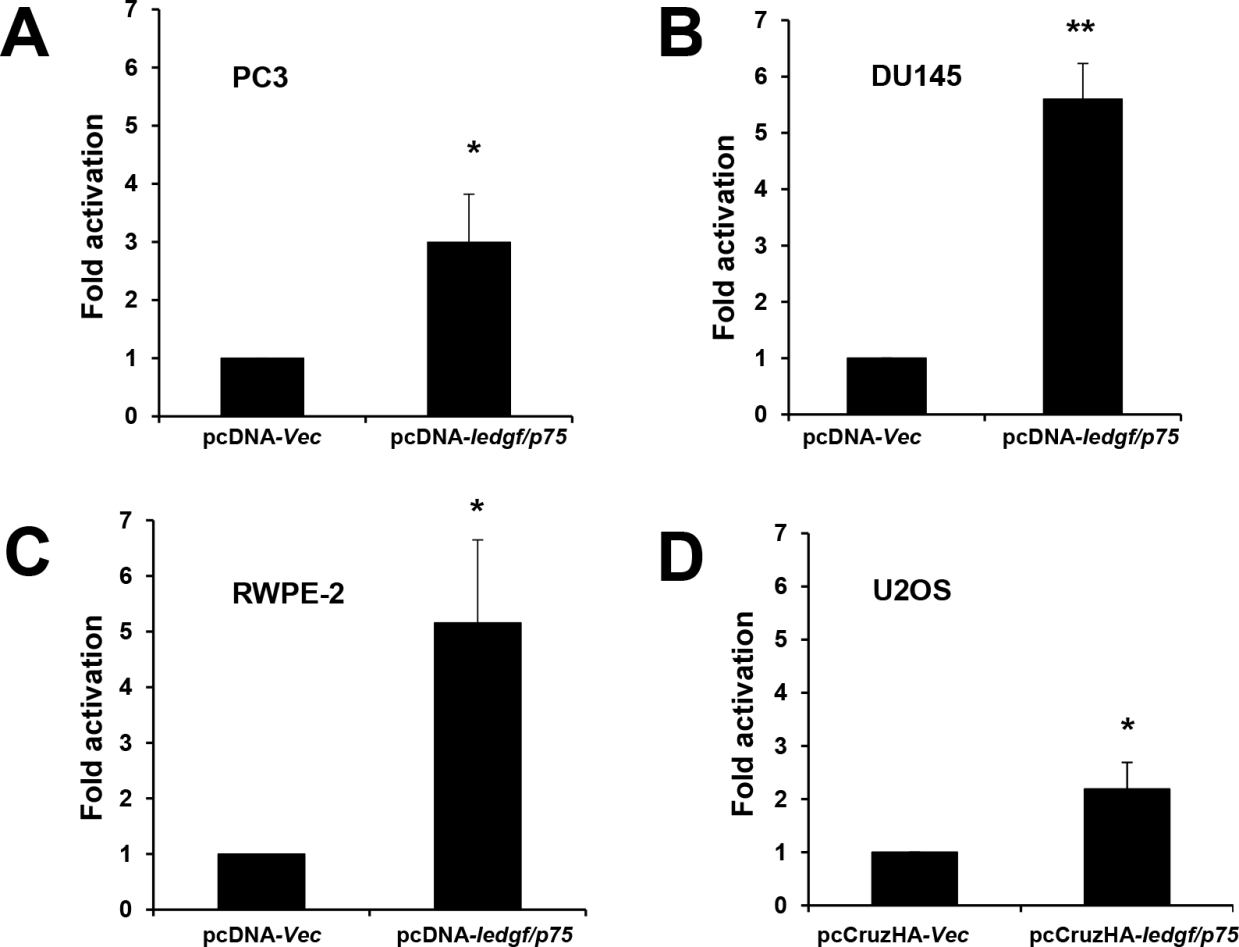
LEDGF/p75 transactivates ERp57 promoter in luciferase-based transcription reporter assays. *ERp57pr* transactivation by LEDGF/p75 in A. PC3; B. DU145; C. RWPE-2; and D. U2OS cells. Luciferase activity in PCa cells co-transfected with *ERp57pr* vector and pcDNA-*ledgfp/75* vector was compared to activity in cells co-transfected with *ERp57pr* vector and pcDNA empty vector (A-C). Luciferase activity in U2OS cells co-transfected with *ERp57pr* vector and pCruzHA-*ledgfp/75* vector was compared to activity in cells co-transfected with *ERp57pr* vector and pCruzHA empty vector (D). Promoter activity determined as luciferase light units/protein is expressed as fold activation compared to control activity, which was normalized to one. Each graph represents the average of at least 3 independent experiments performed in triplicates (*P<0.*05*, **P <0.01). P values were determined using the Student’s *t*-test.

These observations led us to further investigate if LEDGF/p75 plays a direct role in transactivating the ERp57 promoter. To determine this, a luciferase transcription reporter analysis was performed in three PCa cell lines and in U2OS cells. We used U2OS cells in previous studies to evaluate the LEDGF/p75-mediated transactivation of Hsp27 promoter [30,37]. PC3, DU145, and RWPE-2 PCa cells were each co-transfected with the pLightSwitch-*ERp57pr* vector and pcDNA3.1-*ledgf/p75*. These cells displayed significant increase in promoter transactivation, as measured by luciferase activity, when compared to cells co-transfected with empty pcDNA3.1 vector and the pLightSwitch-*ERp57pr* vector. LEDGF/p75 significantly stimulated *ERp57pr* activity to 2.99, 5.6, and 5.15-fold in PC3, DU145 and RWPE-2 cells, respectively (Fig 7A–7C). Transcriptional activation of *ERp57pr* was also observed in U2OS cells transfected with pCruzHA-LEDGF/p75 (2.19-fold), compared to cells transfected with empty pCruzHA vector (Fig 7D). These values are consistent with previously recorded values for Hsp27pr transactivation by LEDGF/p75 [30,37]. Together, these results showed that LEDGF/p75 transactivates *ERp57pr*, consistent with the observation that its overexpression or depletion led to ERp57 transcript upregulation or downregulation, respectively.

**Fig 8:**
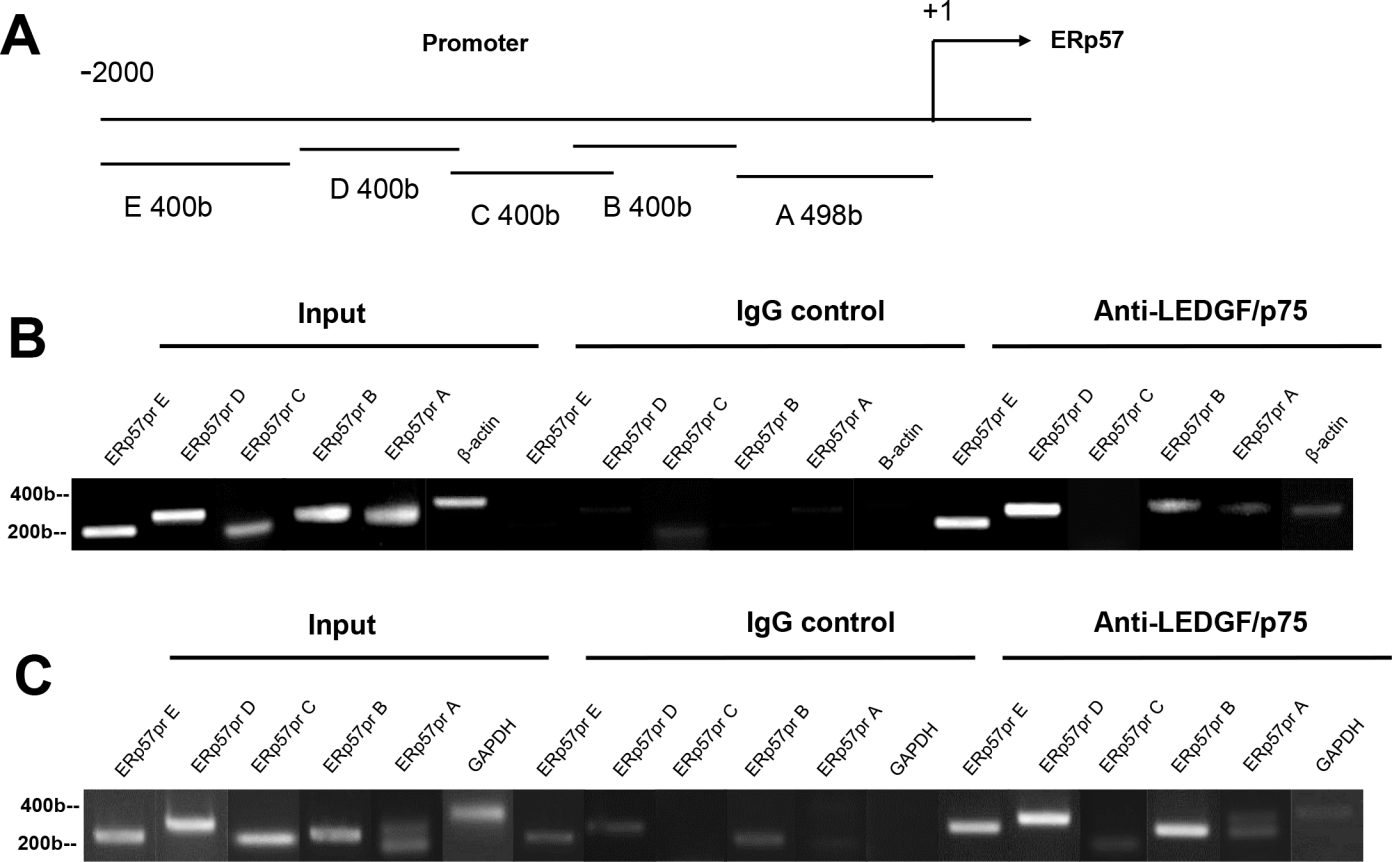
LEDGF/p75 binds to ERp57 promoter in ChIP assays. A. Schematic diagram of ERp57 promoter. PCR primers targeted *ERp57pr* regions A (bp –498 to +1), B (bp –898 to – 490), C (bp – 890 to – 1298), D (bp – 1290 to – 1698), and E (bp – 1690 to – 2098). ChIP analysis of LEDGF/p75 binding to *ERp57pr* in PC3 (B) and U2OS (C) cells. Formaldehyde-fixed cells were precipitated with nonspecific IgG antibody or antibody specific for LEDGF/p75. PCR amplifications of immunoprecipitated DNA were carried out with primer sets specific for *ERp57pr* regions A to E. Primers for human β-actin or GAPDH were used to control for optimal enzymatic digestion of chromatin.

Next we performed ChIP assays to determine if LEDGF/p75 directly binds to the ERp57 promoter region. On the basis of previous studies [27–30], LEDGF/p75 was predicted to bind to regions in *ERp57pr* (bp -2098 to +1) containing stress elements (STRE; consensus A/TGGGGA/T) or heat shock elements (HSE; consensus nGAAn) (Fig 8A). We observed that HSE but not STRE are present at high frequency throughout the entire ERp57 promoter region (data not shown). ChIP assay using a highly specific anti-LEDGF/p75 rabbit polyclonal antibody (Bethyl) revealed binding of this protein, at varying intensities, to *ERp57pr* regions B (bp -898 to -490), D (-1290 to—1698), and E (bp -1690 to -2098) in both PC3 (Fig 8B) and U2OS cells (Fig 8C). Interestingly, the intensity of LEDGF/p75 binding to the individual promoter regions did not correlate with the number of HSE motifs in these regions (data not shown), consistent with the previous observation that binding of this protein to DNA is not restricted to these elements [46]. ChIP with control non-specific IgG antibody did not produce any bands. Negative control primers amplifying GAPDH or β-actin were used to determine the specificity of chromatin precipitation. GAPDH and β-actin were amplified from input DNA but not from DNA precipitated by LEDGF/p75 antibody or control IgG antibody. These results indicated that LEDGF/p75 binds to *ERp57pr*, consistent with the promoter transactivation results.

### Co-expression of LEDGF/p75 and ERp57 proteins in clinical PCa tissues

**Fig 9:**
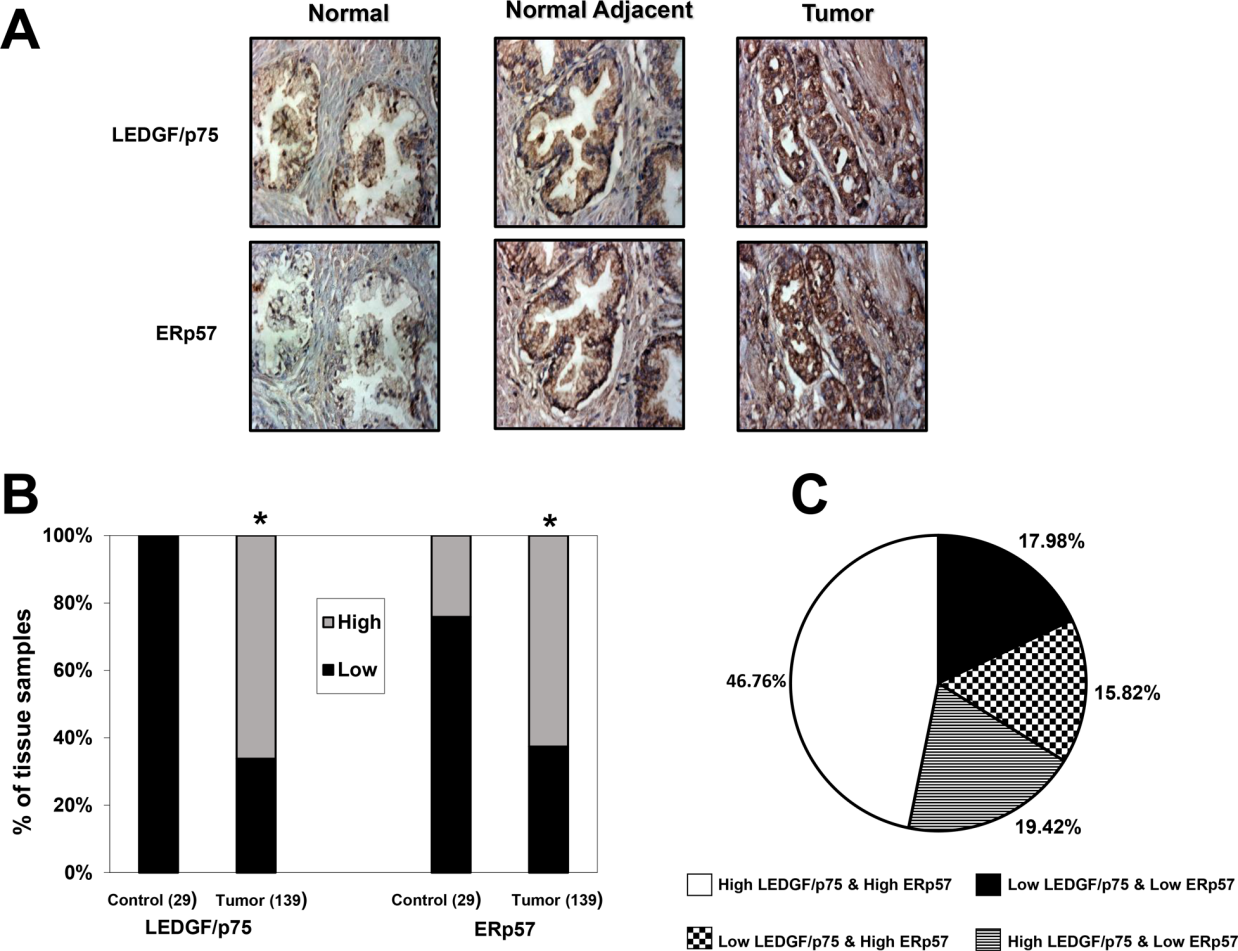
Immunohistochemical analysis of LEDGF/p75 and ERp57 expression in prostate tumor and control tissues. A. Representative images of IHC staining in disease-free normal prostate tissues, matched normal adjacent prostate tissues, and prostate tumor tissues. Images were acquired under identical settings. B. Elevated expression of LEDGF/p75 and ERp57 proteins in tumor tissues compared to pooled controls. Prostate tissue microarray slides were stained with specific antibodies against LEDGF/p75 and ERp57, and the individual tissue cores were scored blindly using the following scale: 0 = no staining, 1 = low staining, 2 = moderate staining, 3 = strong staining. Scored tissues were pooled into two groups: low staining (0–1, dark bars) and high staining (2–3, light bars). The percentage of specimens in the two categories was plotted for tumor tissues compared to control (combined disease-free normal and normal adjacent) tissues. C. Pie chart showing the percentage of tissue specimens with high or low expression levels of LEDGF/p75 and ERp57. *P<0.*05*. *P* values were determined using the Chi-square test.

Since our *in vitro* results indicated that LEDGF/p75 overexpression in PCa cells leads to ERp57 upregulation, and that LEDGF/p75 binds to and transactivates the ERp57 promoter, we hypothesized that the expression of both proteins would correlate in human clinical prostate tumors. To evaluate this hypothesis we performed IHC analysis of LEDGF/p75 and ERp57 protein expression in TMAs containing a total of 140 prostate tumor and 29 control (6 disease-free normal and 23 normal adjacent) tissues. One tumor tissue core could not be scored because of its poor quality. Fig 9A shows representative IHC images of disease-free normal prostate, as well as prostate tumor specimens with their matched normal adjacent tissues, immunostained with anti-LEDGF/p75 and anti-ERp57 antibodies. We observed that the disease-free normal tissues had relatively low-intensity staining of LEDGF/p75 and ERp57. The normal adjacent tissues had moderate staining while the prostate tumor tissues displayed the strongest staining intensity for both LEDGF/p75 and ERp57. The results from the IHC analysis of pooled scores for LEDGF/p75 and ERp57 protein expression (i.e., high = scores 2–3 versus low = scores 0–1) in tumor tissues compared to control tissues are illustrated in Fig 9B. There was a robust overexpression (*P< 0*.*0001*) of both LEDGF/p75 and ERp57 proteins in prostate tumors (N = 139), compared to the control tissues (N = 29).

A correlation analysis between the expression of LEDGF/p75 and ERp57 proteins in the prostate tumor and control tissues revealed significant correlation (*P<0*.*0001*). Our analysis showed that out of 139 prostate tumor tissues, 65 (46.76%) tissues had high expression of both LEDGF/p75 and ERp57, and 25 (17.98%) tumor tissues had low expression of both proteins (Fig 9C). Thus, the expression of these two proteins, whether high or low, significantly correlated in 64.7% of the tumor tissues. However, there were 22 (15.82%) tumor tissue cores with low LEDGF/p75 and high ERp57 protein levels, and 27 (19.42%) tumor tissue cores with high LEDGF/p75 and low ERp57 protein levels, indicating that the correlation between the expressions of these proteins was not complete (Fig 9C). Out of the 29 control prostate tissues, 22 (75.86%) had low expression of both LEDGF/p75 and ERp57, whereas only 7 (24.13%) showed low LEDGF/p75 and high ERp57 expression.

## Discussion

Our results indicate that LEDGF/p75 overexpression attenuates oxidative stress-induced necrosis but not STS-induced apoptosis in PCa cells, consistent with our previous observation that this protein is cleaved during apoptosis by caspase-3 into a p65 fragment that lacks stress survival activity and accelerates cell death [22,23,30]. Interestingly, LEDGF/p75 integrity was preserved during oxidative stress-induced necrosis, which most likely contributed to its protective effects. Converging evidence has linked oxidative stress with lysosomal membrane permeabilization (LMP) and cell death [47]. Under normal conditions, the cellular antioxidant defense system protects against most oxidative events; however, if this protective shield is overwhelmed, ROS induces LMP, cathepsin release, and cell death by either necrosis or apoptosis [47]. Our observation that LEDGF/p75 overexpression reduces intracellular ROS levels in TBHP-treated PCa cells is consistent with previous studies showing that this protein decreases the oxidative stress burden in mammalian cells, resulting in protection of DNA and lysosomes against oxidative damage [7,8,13,14,18,25]. Taken together, these observations support the conclusion that LEDGF/p75 may not act as an inhibitor of apoptosis per se but rather as an upstream antagonist of oxidative stress-induced LMP, which may either occur upstream of apoptosis activation or lead directly to necrosis depending on the cell type and the insult. It remains to be established if LEDGF/p75 protects PCa cells against different types of stressors that induce oxidative stress, particularly antitumor drugs used for PCa treatment that preferentially induce necrosis or programmed necrosis/necroptosis.

While the exact mechanisms by which LEDGF/p75 protects cancer cells against oxidative stress are not entirely clear, compelling evidence suggests that this protein acts as a transcription co-activator and survival factor that augments the expression of stress genes in response to increased microenvironmental stress, leading to enhanced cell survival [8,9,18,25,27–30]. For instance, studies in lens epithelial cells and Cos-7 cells showed that overexpression of LEDGF/p75 induced the transcriptional activation of the antioxidant proteins Hsp27, αB-crystallin, and AOP2/PRDX6, leading to resistance to oxidative damage [7,26–29,48–50]. We also demonstrated previously that transient overexpression of LEDGF/p75 in PCa cells under conditions of stress upregulates a number of stress and antioxidant genes, including cytoglobin, catalase, superoxide dismutase 3, and thyroid peroxidase [24].

LEDGF/p75 overexpression in cancer, with its ensuing transactivation of stress genes in response to microenvironmental stressors, would likely translate into increased tumor cell survival and resistance to therapy. This notion has been validated in numerous studies demonstrating a role for overexpressed LEDGF/p75 in promoting increased cell proliferation, clonogenicity, invasion, migration, angiogenesis, chemoresistance, and tumor volume in pre-clinical models of PCa and other cancers, whereas its depletion or inactivation causes decreased cell survival and tumor growth [12–14,16–22,30]. However, LEDGF/p75 may not be essential for cell survival, given that cancer cell clones with stable depletion of this protein can be successfully developed (this study and refs. [24,51]), and that PSIP1/LEDGF/p75^-/-^ knockout mice can survive, albeit with multiple skeletal malformations leading to premature death [52]. While these conflicting observations are likely to be cell-type or context dependent, it is plausible that LEDGF/p75-depleted clones may have developed compensatory survival mechanisms, allowing for cell survival in the absence of this protein [24].

Our results demonstrated that overexpression of LEDGF/p75 in PCa cells induced the transactivation and upregulation of ERp57, consistent with its function as a stress response transcription co-activator. This is supported by the following observations: 1) LEDGF/p75 overexpression leads to ERp57 transcript and protein upregulation in PCa cells, 2) LEDGF/p75 depletion leads to decreased ERp57 transcript expression in PCa cells, 3) LEDGF/p75 binds to and transactivates the ERp57 promoter region in PCa cells, and 4) LEDGF/p75 expression correlates with ERp57 expression in 65% of PCa tissues and 76% of control prostate tissues.

ERp57 is a member of the protein disulfide isomerase family that is mainly located in the endoplasmic reticulum, and has well-characterized functions as a cellular protective protein [31,32,53]. It participates in the quality control of newly synthesized glycoproteins, in association with calnexin/calreticulin, and is also an important component of the major histocompatibility complex class I peptide loading complex [53–55]. ERp57 also acts as a cellular redox sensor to adapt cells to oxidative insults, and regulates protein-protein interactions via a redox mechanism using its two thioredoxin-like domains [56]. Its overexpression results in a marked protective effect against hydrogen peroxide-induced cell death [56].

The role of ERp57 in tumor progression is still unclear. While some studies revealed that its overexpression correlates with cellular invasiveness and bone metastasis in cervical and breast cancer, respectively [57,58], one study demonstrated that loss of this protein is associated with more aggressive gastric cancer [59]. ERp57 was identified as a chemoprevention target in colon cancer cells [60], and as a biomarker of taxol chemoresistance and radioresistance in ovarian and laryngeal cancers, respectively [61,62]. Its elevated expression was also reported in lung cancer and ovarian cancer cells [63,64].

We observed a significant correlation between the expression of LEDGF/p75 and ERp57 proteins in clinical prostate tumor tissues, as detected by IHC. This correlation was facilitated by the elevated expression of both proteins in prostate tumor tissues as compared to their relatively low levels in control tissues. While the upregulation of LEDGF/p75 in prostate tumors is consistent with our previous reports [11,23], the observed ERp57 upregulation in these tumors (62% of prostate tumor tissues versus 24% of control tissues) appears to be inconsistent with a previous study showing downregulation of this protein in PCa tissue compared to normal adjacent tissue [65]. This apparent discrepancy could be related to differences in patient cohorts, control tissues, staining platforms, or IHC scoring criteria. It should be noted, however, that a more recent report revealed upregulation of ERp57 in prostate tumors as compared to benign prostatic tissue using 2D-DIGE coupled with mass spectrometry [66]. ERp57 expression was also found upregulated in prostate tumors with high Gleason scores in a study that compared tissue samples from low- and high-risk prostate tumors [67]. The mechanisms underlying ERp57 upregulation in prostate tumor tissues are not clear, but it is plausible that the high metabolic rate of PCa cells, combined with the pro-inflammatory prostate tumor microenvironment, increases oxidative stress leading to upregulation of stress and antioxidant defense proteins such as LEDGF/p75 and its target genes [68].

A puzzling observation in our studies was that although depletion of LEDGF/p75 in PCa cells resulted in downregulation of ERp57 transcript expression, it failed to downregulate ERp57 protein expression. This lack of transcript-protein correlation could be attributed to various factors including abnormal ERp57 protein stabilization via protein-protein interactions, post-translational modifications, differential lifetimes of mRNA and protein species, mRNA regulation by miRNAs, differential regulation or processing of protein isoforms, and other mechanisms not yet completely understood [69,70]. Indeed, several integrative studies of transcript and protein expression profiles in tumor tissues and cancer cell lines have described this discordant mRNA-protein correlation for other cancer-associated proteins [70–74]. There is also precedent for the observation that while overexpression of LEDGF/p75 can induce the upregulation of a particular stress gene, depending on the cell type its depletion may not necessarily cause changes in the expression of the same gene [17,24,75].

A likely explanation for the discordant ERp57 transcript-protein expression in response to changes in LEDGF/p75 expression could be that because of its role as a chaperone protein, ERp57 may engage in protein-protein interactions that increase its stability and long half-life in PCa cells. Another possibility is that residual levels of LEDGF/p75 protein left in the siRNA-depleted cells might still be sufficient for induction of basal ERp57 transcript and protein. It is also plausible that basal expression levels of ERp57 in PCa cells are controlled by other transcription factors, and that the role of LEDGF/p75 is to enhance the transcription of this and other stress proteins (e.g. Hsp27, PRDX6) only when the cancer cells need to develop a stress survival response in the presence of a stressful or pro-inflammatory microenvironment characterized by unhealthy levels of oxidative stress. Thus, while LEDGF/p75 would play an important role in the upregulation of stress protective genes as part of the cellular stress response, its depletion may not necessarily impact the expression of these genes under basal conditions.

In summary, this study provides further evidence that LEDGF/p75 is a stress survival oncoprotein that when overexpressed protects PCa cells from oxidative stress-induced necrosis, most likely by augmenting the transcriptional activation of specific stress survival, cancer-associated proteins. Recently, we proposed a model in which LEDGF/p75 overexpression in the context of a pro-inflammatory or stressful microenvironment may not only contribute to increase cell survival in stressed tissues, including tumors, but also trigger a robust IgG autoantibody response to this protein in genetically susceptible individuals [9]. Further studies are required to determine if ERp57 upregulation is essential for the protective effects of LEDGF/p75, particularly in the context of tumor aggressiveness and resistance to therapy. LEDGF/p75 and ERp57 might be important components of the mechanism of resistance developed by prostate tumors against therapeutic modalities that induce oxidative damage and cell death such as chemotherapy or radiation, and could represent promising targets for combinatorial therapies for aggressive PCa.
